# Astrocytic β2 Adrenergic Receptor Gene Deletion Affects Memory in Aged Mice

**DOI:** 10.1371/journal.pone.0164721

**Published:** 2016-10-24

**Authors:** Cathy Joanna Jensen, Frauke Demol, Romy Bauwens, Ron Kooijman, Ann Massie, Agnès Villers, Laurence Ris, Jacques De Keyser

**Affiliations:** 1 Department of Neurology, University Hospital Brussels and Center for Neuroscience, Vrije Universiteit Brussel (VUB), Brussels, Belgium; 2 Department of Neurobiology, Vlaams Instituut voor Biotechnologie, Leuven, Belgium; 3 Center for Neurosiences, Vrije Universiteit Brussel, Brussels, Belgium; 4 Department of Neuroscience, Health, University of Mons, Mons, Belgium; 5 Department of Neurology, University Medical Center Groningen, Groningen, The Netherlands; Centre de Recherche Jean-Pierre Aubert, FRANCE

## Abstract

*In vitro* and *in vivo* studies suggest that the astrocytic adrenergic signalling enhances glycogenolysis which provides energy to be transported to nearby cells and in the form of lactate. This energy source is important for motor and cognitive functioning. While it is suspected that the β2-adrenergic receptor on astrocytes might contribute to this energy balance, it has not yet been shown conclusively *in vivo*. Inducible astrocyte specific β2-adrenergic receptor knock-out mice were generated by crossing homozygous β2-adrenergic receptor floxed mice (*Adrb2*^*flox*^) and mice with heterozygous tamoxifen-inducible Cre recombinase-expression driven by the astrocyte specific L-glutamate/L-aspartate transporter promoter (*GLAST-CreERT2*). Assessments using the modified SHIRPA (SmithKline/Harwell/Imperial College/Royal Hospital/Phenotype Assessment) test battery, swimming ability test, and accelerating rotarod test, performed at 1, 2 and 4 weeks, 6 and 12 months after tamoxifen (or vehicle) administration did not reveal any differences in physical health or motor functions between the knock-out mice and controls. However deficits were found in the cognitive ability of aged, but not young adult mice, reflected in impaired learning in the Morris Water Maze. Similarly, long-term potentiation (LTP) was impaired in hippocampal brain slices of aged knock-out mice maintained in low glucose media. Using microdialysis in cerebellar white matter we found no significant differences in extracellular lactate or glucose between the young adult knock-out mice and controls, although trends were detected. Our results suggest that β2-adrenergic receptor expression on astrocytes in mice may be important for maintaining cognitive health at advanced age, but is dispensable for motor function.

## Introduction

Astrocytes are the main cellular targets of norepinephrine (NE) terminals in the brain [1] and the β_2_AR is present on astrocytes in mice, along with the α_1A_, α_2A_ and β_1_ adrenoreceptors [2]. Detailed *in vitro* and *in vivo* studies using subtype-selective agonists and antagonists have shown that signaling through the β_2_ adrenergic receptor (β_2_AR) influences a range of CNS processes including metabolic activity, inflammation, glutamate and potassium buffering, and many other functions [3], [4–6]. *In vitro* studies have shown that some of the effects of stimulation of the β_2_AR on astrocytes are mediated by elevation of intracellular cAMP levels, leading to PKA activation. A shift from nuclear factor kappa B pathway (NF-κB) to the peroxisome proliferator activated receptor gamma (PPAR-γ) pathway also occurs, leading to the release of different neurotrophic factors, cytokines, chemokines and nitric oxide (NO).

During neural activity, NE stimulation influences potassium homeostasis and enhances astrocytic glutamate uptake from the synaptic cleft, thereby preventing the overload of glutamate and subsequent intracellular Ca^2+^ elevation, a condition known as excitotoxicity [4, 7, 8]. These processes are essential for brain function, but are very energy expensive. Thus, is makes sense that NE is also involved in the regulation of energy in the CNS [9–11]. The regulation of energy by NE is tightly linked to the metabolic needs of glutamate and potassium homeostasis during neuronal activity which must be maintained both inside and outside cells, mostly by astrocytes which are equipped by both passive and active uptake capabilities [4, 7]. Astrocytes are the main, if not only, store of glycogen in the central nervous system, and provide and energy source not only for the function of astrocytes, but also for neurons and potentially other cell types [7, 12–14]. Regulation of glycogen metabolism by NE is shared between the adrenergic receptor subtypes; α_2_AR stimulates a net increase of glycogen in the astrocyte, primarily due to a reduction in cAMP while stimulation of the β receptors enhances the breakdown of glycogen in astrocytes, leading to lactate production and release by means of the glycogen shunt [4] [15].

Whole body knockouts of the β2AR have been studied for some time, however the receptor has a broad range of effects on peripheral body systems, and we were interested in what effect the specific astrocytic loss might have on motor and cognitive function. For this reason, we developed an inducible astrocytic knockout of β_2_AR by crossing a mouse with floxed β_2_AR [16] with a mouse expressing CreERT2 under the glutamate aspartate transporter (*GLAST*, also *SLC1A3*) promotor [17]. While *GLAST* is expressed in some cell types outside of the CNS [18], within the CNS in adult mice, the expression is considered specific to astrocytes and radial glial cells at early stages of development, along with those rare cells undergoing neurogenesis [17].

## Materials and Methods

### Animals

All mice used were bred and kept under standard housing conditions with a 12 h dark/light cycle and with food and water *ad libitum* in our facilities. All experiments were carried out in accordance with the National Rules on Animal Experiments and were approved by the Ethics Committee on Animal Experiments of the Vrije Universiteit Brussel.

Conditional, inducible transgenic mice were generated by breeding *Adrb2*^*flo*x^ mice [19] with GLAST-CreERT2 [17] mice, to generate *Adrb2*^*flo*x^ GLAST-CreERT2 double mutants on a C57Bl/6 background. Specific ablation of the *Adrb2* gene was induced in GLAST expressing cells at 8 weeks of age via 5 consecutive days of twice daily intraperitoneal injections of 1mg tamoxifen dissolved in corn oil [17]. Four groups were included in these experiments. Genotype groups used in these experiments were: homozygous *Adrb2*^*flo*x^ and heterozygous GLAST-CreERT2 (*Adrb2*^*flox+/+*^ *GLAST-CreERT2*^*+/-*^*)* animals treated with tamoxifen (KO) or the corn oil vehicle (CO), tamoxifen treated conditional Cre expressing mice (CRE) (*Adrb2*^*flox-/-*^*GLAST-CreERT2*^*+/-*^) and tamoxifen treated floxed mice (FLX) (*Adrb2*^*flox+/+*^*GLAST-CreERT2*^-/-^). Mice were assessed at 1, 2 and 4 weeks, 6 and 12 months after the induction of genetic recombination. Other mice of the same colony were assessed at advanced age (18 months). Some mice were found dead before this age, but no signs of illness, stress or morbidity were observed that were associated with the genetic treatment.

### Genotyping

Genomic DNA was extracted from mouse tail clips using and genotyping was performed using the following primers for GLAST-CreERT2 genotyping: GLAST F8 (5’-AGGCACTTGGCTAGGCTCTGAGGA-3’), GLAST R3 (5’-GAGGAGATCCTGACCGATCAGTTGG-3’) and CER1 (5’-GGTGTACGGTCAGTAAATTGGACAT-3’) as previously described by Mori et al [17]. The presence of the *Adrb2*^flox^ gene was assessed with the primers *Adrb2* F (5’-AGCTGAGTGTGCAGGACGCA-3’) and *Adrb2* R (5’-CGCTTCGTCCCGTTCCTGAGT -3’) [19].

### MACS separation

Mice were sacrificed by injection of an overdose of sodium pentobarbital (Ceva, Nembutal 60 mg/ml). Whole brains of 10–12 week old mice were dissociated manually using a commercial neural tissue dissociation kit (Milteyni Biotec). GLAST (ACSA-1) positive cells were collected from the single cell suspensions using the MACS system (Miltenyi Biotec, catalog #130-095-826) as described by the manufacturer. Cells from each animal were isolated and analyzed separately.

### RNA isolation and qPCR

RNA was isolated from the GLAST positive cell pellet using TRIzol (Life Technologies), cDNA was reverse transcribed and the expression of transcripts for *Adrb2* and *Gapdh* were assessed using the primer sets *Adrb2* forward 5'-TGTTACACCGAGGAGACTTGCTGTG-3' and reverse 5'-ACCACCAGGGGCACGTAGAAA-3' and *Gapdh* forward 5'-CAAGGCTGTGGGCAAGGTCATC-3' and reverse 5'-GGGGGTAGGAACACGGAAGG-3' with SYBR green technology (Life Technologies).

### Phenotyping

Males and females from each experimental group were investigated in this study (n = 10). In short, the phenotyping consisted of 3 separate tests: the modified SHIRPA, the swimming ability test and the accelerating rotarod test. Phenotyping analysis was performed at 1, 2 and 4 weeks, 6 and 12 months after tamoxifen induction. To avoid bias due to learning by repeated testing, different animals were used for the tests on 1, 2 and 4 weeks after induction. Before testing, the animals were left to habituate in the testing room for a minimum of one hour.

We performed the modified SHIRPA testing [20] as described in the European Mouse Phenotyping Resource of Standardized Screens (EMPReSS) protocol (http://empress.har.mrc.ac.uk/browser/). Briefly, animals were weighed and placed in a clear cylindrical viewing jar where body position, tremor, palpebral closure, appearance of the coat, whiskers, lacrimation and defecation were scored. Afterwards the mouse was dropped into an arena from a height of approximately 25 cm. Transfer arousal, locomotor activity, gait, tail elevation and touch escape were assessed. Finally positional passivity, skin colour, trunk curl, limb grasping and evidence of biting and vocalization were recorded.

Swim ability testing was performed as described by the EMPReSS protocol. Mice were lowered carefully in the cylindrical viewing jar containing 15 cm of water at 24–26°C allowing free movement and swimming of the mouse. Mice were observed for one minute and swimming ability was assessed according to the following scores 0 = normal swimming, 1 = irregular swimming, 2 = immotile floating and 3 = under water tumbling.

The rotarod test was performed as described in the EMPReSS database, with minor modifications (TSE systems, RotaRod System Advanced, 3375-R4). In the training phase, animals had 4 sessions on the rotarod with an inter-training interval of 10 minutes. The first session consisted of 1 min on the non-rotating rod followed by 1 min on the rod with a constant speed of 4 rpm. The 3 following training sessions consisted of 1 min at a constant speed of 4 rpm. Animals that were not able to sustain 1 minute at 4 rpm during the training phase were excluded from the test phase. Between training and testing a 30 minute break was implemented. In the test phase, four sessions were performed in which the rod accelerated from 4 rpm to 40 rpm in 300 sec. The inter-test interval was 15 min. Latency time to fall of the rod was recorded.

### Morris water maze

Mice in the young group were assessed 4 weeks after the induction of genetic recombination (12.±1.0 weeks of age) and no differences was observed in their body weights (KO N = 5, CO N = 5, Cre N = 8, FLX N = 9), All were males.

In the aged animal groups, vehicle control mice at 63 weeks (N = 3) and 75 weeks (N = 3) were combined in one control group after having confirmed the absence of differences in the escape latency or path length in the visible or hidden phases, or in the probe phase (S2 Fig)

In the main experiment the CO aged mice were older than the KO mice (69.2±6.3 weeks, N = 6 compared to 60.6± 1.0 weeks, N = 5 respectively) and their body weight were slightly more (46.2±5.8g compared to 37.8± 4.1g respectively). All were males.

The apparatus for the water maze was located in a single-purpose laboratory room and consisted of a circular pool made of black plastic (diameter, 150 cm; height, 100 cm) filled with water (20–22°C) opacified with a nontoxic white paint. A Plexiglas platform, 10 cm in diameter and 40cm high with a textured surface was placed at one of 5 positions in the maze. The maze was filled with opacified water (20–22°C) to a depth that was 0.5cm below the top of the platform for the visible platform training, and 0.5cm above the platform for hidden platform trials. The apparatus was surrounded on four sides by white curtains which were 30cm distant from the pool wall at their closest point. Distal spatial cues (coloured shapes with bold patterns) were attached to the curtains at a height where all 4 cues could be seen from the centre of the pool. The 12 week old animals were trained for 2 days with the visible platform) and 5 days with the hidden platform (four trials per day of 1 minute followed by, 15 seconds on the platform, with a 30 minute inter-trial duration. The aged animals underwent a less taxing protocol. Animals were trained for 2 days with a visible platform (three trials per day of 1 minute followed by 15 seconds on the platform, 30 minute inter-trial duration) and then 9 days of the hidden platform before the probe (three trials per day of 1 minute, 15 seconds on the platform, 30 minute inter-trial duration). The start position was randomized for all mice. Escape latency, escape path, swimming speed and time spent in each virtual quadrants of the maze were recorded and analyzed using Noldus Ethovision 3.0 tracking system.

For all mice, two hours after the last training session on the last day, a probe trial was performed with the platform removed from the pool. The animals were introduced in the quadrant opposite to the target quadrant and allowed to search for the platform for 30 s. Spatial acuity was expressed as the percentage of time spent in each of the four virtual quadrants and spatial memory was assessed by the time spent in the target quadrant.

### Long term potentiation

Transverse hippocampal slices (400-μm thickness) were prepared as described by Nguyen and Kandel (1997). The hippocampus was isolated and sliced with a McIlwain chopper. Slices were allowed to recover for 1.5 h at 28°C in interface in aCSF of the following composition: 124 mM NaCl, 5 mM KCl, 26 mM NaHCO3, 1.0 mM NaH2PO4, 2.5 mM CaCl2, 1.3 mM MgSO4, 10 mM glucose. The aCSF was aerated with 95% O2 and 5% CO2. After this recovery period, the glucose concentration was either left unchanged or lowered to 2 mM. In the latter case, sucrose was added for osmotic stability. All the recordings were made in an interface chamber (FST) at 28°C. The rate of flow of the perfused liquid was 1 mL/ min. Baseline recording started after 15 min and lasted for 30 min. LTP was induced electrically by applying a single 1-sec train (100 Hz, at test strength). Extracellular fEPSPs were recorded with a glass microelectrode (2–5 Mohm, filled with aCSF) positioned in the stratum radiatum of area CA1. A bipolar nickel–chromium stimulating electrode was used to elicit fEPSPs by stimulation of the Schaffer collateral fibers. For each slice, an input/output curve was recorded by increasing stimulation intensity from 2 to 10 V. Afterwards, stimulation intensity (0.08-msec pulse duration) was adjusted to elicit fEPSP amplitudes that were ∼40% of maximum size. Basal synaptic transmission was assessed by stimulating Schaffer collaterals once per minute at this test stimulation intensity.

### Microdialysis

Surgery: Male mice 4 weeks post induction of all four groups, weighing 29.97 ± 2.423g, were anesthetized with a mixture of xylazine/ketamine (10/100 mg/kg, i.p.) and mounted on a stereotaxic frame. An intracranial guide (CMA/Microdialysis, Stockholm, Sweden) was implanted in the bilateral cerebellar white matter (Coordinates towards bregma were 5.7 mm posterior, 2.2 mm lateral and 2.3 mm ventral). Immediately after surgery, guide cannula obturators were replaced by microdialysis probes (CMA7; membrane length: 1 mm theoretical cutoff: 6000 Da; CMA/Microdialysis, Solna, Sweden). Postoperative mice received a subcutaneous injection of 0.2 ml saline in both flanks. Animals were allowed to recover from surgery overnight and received laboratory chow and water ad libitum. Probe localization was histologically verified postmortem with cresyl violet staining. Animals with aberrant probe location were omitted from the analysis.

Intracerebral Microdialysis: Microdialysis probes were continuously perfused with aCSF (147 mM NaCl, 3 mM KCl, 1 mM MgCl2.6H_2_O, 0.787 mM CaCl_2_.6H_2_O, 186 μM Ascorbate, 3.52 mM NaH_2_PO_4_.H_2_O, pH 7.4) at the work flow-rate of 1 μl/min (CMA/400 microdialysis pump). All experiments were performed on the day following surgery. Tubing for the right probe was flushed with 70% ethanol and rinsed with purified water before perfusion with aCSF to exclude any bacterial interference. 1 μl/min perfusion of the right probe was started one hour before the experiment to attain steady-state concentrations of the substrates. Eight dialysate samples (20 μl) were collected at 20-minute intervals.

### CSF glucose and lactate assays

Enzymatic lactate (K607-100) and glucose (K606-100) assay kits were purchased from Biovision (Mountainview, Ca, U.S.A.). Microdialysate samples were analyzed by a colorimetric assay for glucose and lactate content according to the manufacturers’ guideline. Optic density at 570 nm was measured with a microplate reader (Model 680 Bio-Rad, Hercules, CA, USA).

### Blood glucose

Mice were fasted for 5–6 hours and then blood was collected from tail clips. Blood glucose was measured with Accu-check Aviva glucose strips (Roche) and blood glucose meter according the manufacturer’s instructions.

### Statistics

All categorical behavioral tests were assessed for significant differences with the two-proportion test, comparing each genotype group to the combined population at that age group followed by the post hoc z-test with Bonferoni correction at each time point. Scale variables (weight, locomotor activity and rotarod) were tested for significant differences with Kruskall-Wallis test and Dunn’s multiple comparison post hoc test.

In longitudinal studies, tests for significant differences were done with two way ANOVA (genotype, time point) followed by Bonferroni multiple comparison test if appropriate. The quadrant test was assessed with one way ANOVA, followed by Tukey’s multiple comparison test on time spent in the target quadrant where appropriate. When only one variable was tested, Student’s *t* test was used. The alpha value was set at 0.05 for each statistical test.

## Results

### Generation of an inducible selective astrocytic β2AR knock out mouse model

**Fig 1:**
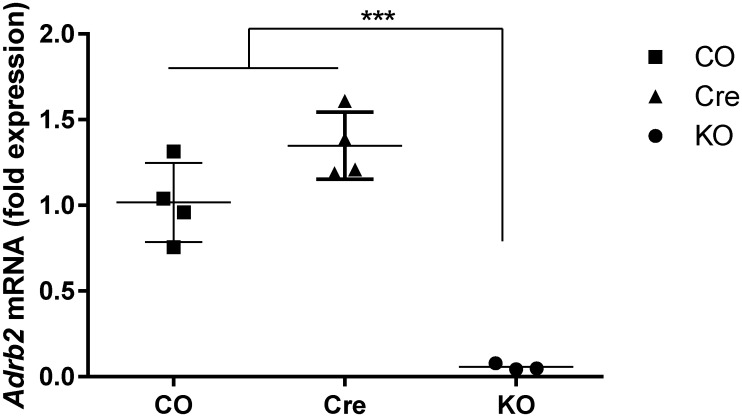
ADRB2 mRNA expression in MACS-isolated astrocytes from *Adrb2*^*flo*x^ GLAST-CreERT2 double mutants and controls. Genotype groups used were: *Adrb2*^*flo*x +/+^ GLAST-CreERT2^+/-^ animals treated with tamoxifen (KO) or the corn oil vehicle (CO), tamoxifen treated conditional Cre expressing mice (Cre) (*Adrb2*^*flo*x -/-^GLAST-CreERT2^+/-^) (N = 4).

The breeding strategy described in the materials and methods section resulted in double mutant *Adrb2*^*flox*^*GLAST-CreERT2* mice. Litter size was normal for C57BL/6 mice (6.12 ± 2.29) and both males and females were fertile (data not shown). Mice appeared to be generally healthy and there were no visible malformations. At 8 weeks of age mice received tamoxifen to induce the ablation of the β_2_AR in GLAST expressing cells. Cell specific knock down of the β_2_AR transcript was confirmed by enriching for GLAST expressing cells from the brain using MACS followed by qPCR. Results indicate a highly significant (p<0.001) 20-fold down regulation of β_2_AR mRNA in the KO model compared to vehicle-only control (N = 5). *Adrb2* gene expression was not affected by tamoxifen or Cre expression as mRNA expression was unchanged in astrocytes isolated from tamoxifen-treated Cre mice compared to vehicle-only CO controls (p = 0.071) (Fig 1).

### Body weight and temperature

**Fig 2:**
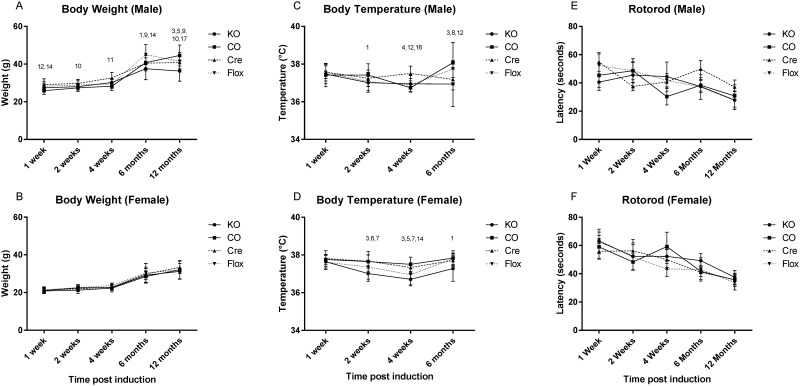
General phenotyping for *Adrb2*^*flo*x^ GLAST-CreERT2 double mutants. Genotype groups used were: *Adrb2*^*flo*x +/+^ GLAST-CreERT2^+/-^ animals treated with tamoxifen (KO) or the corn oil vehicle (CO), tamoxifen treated conditional Cre expressing mice (CRE) (*Adrb2*^*flo*x -/-^GLAST-CreERT2^+/-^) and tamoxifen treated floxed mice (FLX) (*Adrb2*^*flo*x +/+^GLAST-CreERT2^-/-^). The body weight of (A and B), body temperature (C ad D), and latency to fall on the RotaRod (E and F) is shown at time points after the tamoxifen treatment. Rotorod data is shown as the mean +/- SEM, other measure are mean +/- SD. Significance: 1 KO+ vs CO p<0.05, 2 KO+ vs CO p<0.01, 3 KO+ vs CO p<0.001, 4 KO+ vs CRE p<0.05, 5 KO+ vs CRE<0.01, 6 KO+ vs CRE p<0.001, 7 KO vs FLX p<0.05, 8 KO vs FLX <0.01, 9 KO vs FLX p<0.001, 10 CO vs CRE p<0.05, 11 CO vs CRE<0.01, 12 CO vs CRE p<0.001, 13 CO vs FLX p<0.05, 14 CO vs FLX<0.01, 15 CO vs FLX p<0.001, 16 CRE vs FLX p<0.05, 17 CRE vs FLX<0.01, 18 CRE vs FLX p<0.001.

Body weight was assessed at 1, 2 and 4 weeks, 6 and 12 months after the tamoxifen-induction of genetic recombination of *Adrb2*. Both male and female animals gained weight normally with age which accounted for 12.46 and 73.36% of the variability in weight gain respectively, but genotype was also a factor in the females (F = 2.89, p = 0.0356), contributing to the variance by 0.63%, possibly an artifact due to the low variance in these animals. No differences were found in pairwise post hoc tests however (Fig 2A and 2B). The body weight of males was not affected by genotype overall (p = 0.6).

The body temperature of mice was assessed 1, 2 and 4 weeks, and 6 months after the induction of genetic recombination. Some differences were seen in female mice when assessed by 2-way ANOVA (age, genotype), with genotype having a significant overall effect on body temperature (F = 19.76. p<0.001). In post hoc analysis, KO mice had slightly reduced body temperature compared to other groups 2 and 4 weeks after induction. FLX mice also had reduced body temperature at 4 weeks after induction compared to Cre and CO mice. Male mice did not show the same course of temperature changes as the females. Some differences were observed, but the variation did not appear to be linked to the genetic recombination or Cre expression (Fig 2C and 2D).

### Astrocytic *Adrb2* loss does not affect general health

General phenotype analysis was performed at 1, 2 and 4 weeks, 6 and 12 months after tamoxifen induction. Minimal sample size at each different time point was 20 mice per genotype. Each genotype group included at least 10 animals per sex. No differences between males and females were identified and thus the data from male and female mice were combined with the exception of the rotarod test, in which females routinely outperform males in laboratory studies (Fig 2E and 2F)

No general health phenotype was observed using the Modified SHIRPA screen at any time points up to 12 months post tamoxifen induction using the 2-propotiton test with Bonferroni correction for multiple testing (Table 1).

**Table 1:** Modified SHIRPA phenotyping and swimming ability results. Results are presented in percentages unless otherwise indicated. No significant differences in parameters were identified for any genotype at any age point using the 2-proportion test with Bonferroni correction. Trends with P-val <0.10 are indicated (*).Genotypes shown are: *Adrb2*^*flo*x +/+^ GLAST-CreERT2^+/-^ animals treated with tamoxifen (KO+) or the corn oil vehicle (CO), tamoxifen treated *Adrb2*^*flo*x -/-^GLAST-CreERT2^+/-^ animal (CRE) and tamoxifen treated *Adrb2*^*flo*x +/+^GLAST-CreERT2^-/-^ mice (FLX).

		1 Week				2 weeks				4 Weeks				6 Months				12 Months			
		KO	CO -	CRE	FLX	KO	CO -	CRE	FLX	KO	CO -	CRE	FLX	KO	CO -	CRE	FLX	KO	CO -	CRE	FLX
	N	25	27	22	25	24	27	27	26	26	24	29	27	26	24	22	21	20	14	20	21
*Neurologic signs*																					
tremor	Present	36.0	11.1	18.2	12.0	41.7	31.6	22.2	15.4	19.2	34.8	18.2	18.2	33.3	52.0	27.3	23.8	45.5	40.0	40.0	19.0
coat	Abnormal	28.0	0.0	9.1	0.0	4.2	0.0	29.6	3.8*	15.4	0.0	9.1	13.0	58.3*	48.0	45.5	28.6	59.1	56.0	35.0	28.6
whiskers	Absent	12.0	0.0	0.0	0.0	20.8	0.0	0.0	0.0	15.4	17.4	0.0	4.3	54.2	44.0	9.1	0.0	50.0	48.0	0.0	0.0
Palpebral closure	Open	100	100	100	100	100	100	100	100	100	100	100	100	100	100	100	100	100	100	100	100
Lacrimation	Absent	100	100	100	100	100	100	100	100	100	100	100	100	100	100	100	100	100	100	100	100
*Handlling related bebaviousr*																					
Transfer arousal	< 1 sec	48.0	63.0	36.4	40.0	50.0	45.9	40.7	38.5	26.9	17.4	45.5	34.8	25.0	28.0	36.4	38.1	50.0	44.0	20.0	23.8
	1–5 sec	32.0	25.9	40.9	28.0	33.3	24.3	29.6	34.6	46.2	43.5	22.7	34.8	37.5	44.0	27.3	28.6	22.7	40.0	40.0	28.6
	>5 sec	20.0	11.1	22.7	32.0	16.7	29.7	29.6	26.9	26.9	39.1	31.8	30.4	37.5	28.0	36.4	33.3	27.3	16.0	40.0	47.6
grasp reflex	Absent	0.0	3.7	0.0	0.0	0.0	0.0	0.0	0.0	0.0	0.0	0.0	0.0	0.0	0.0	4.5	0.0	4.5	4.0	0.0	0.0
biting	Present	12.0	14.8	13.6	24.0	15.4	26.1	22.7	26.1	15.4	26.1	22.7	26.1	16.7	24.0	22.7	19.0	0.0	4.5	10.0	4.8
vocalisation	Present	28.0	7.4	18.2	44.0	19.2	17.4	13.6	17.4	19.2	17.4	13.6	17.4	12.5	20.0	9.1	9.5	4.5	4.0	0.0	14.3
defecation		8.0	29.6	9.1	8.0	8.3	21.1	14.8	7.7	15.4	4.3	18.2	0.0	12.5	28.0	18.2	19.0	9.1	16.0	35.0	28.6
*Motor Skills*																					
locomotor activity	sec	11.8 ± 1.5	12.2 ± 1.2	9.1 ± 1.1	5.9 ± 1.0	9.0 ± 1.0	8.9 ± 1.2	7.6 ±1.1	8.1 ± 1.2	8.4 ± 1.2	8.4 ± 1.2	9.3 ± 1.5	6.8 ± 1.1	6.9 ± 1.2	8.8 ± 1.2	8.2 ± 1.2	6.7 ± 1.1	7.0 ± 0.9	9.6 ± 1.2	7.2 ± 1.2	4.4 ± 0.7
Gait		36.0	18.5	22.7	28.0	16.7	18.9	11.1	30.8	30.8	30.4	18.2	13.0	37.5	24.0	31.8	28.6	54.5	28.0	26.3	42.9
Trunk curl	absent	100	100	100	100	100	100	100	100	100	100	100	100	100	100	100	100	100	100	100	100
Spontaneous activity	Hyper	0.0	0.0	0.0	0.0	0.0	0.0	0.0	0.0	0.0	4.3	0.0	0.0	4.2	0.0	4.5	0.0	0.0	4.0	5.0	0.0
	active	72.0	92.6	90.9	52.0	66.7	89.5	85.2	88.5	69.2	69.6	95.5	78.3	70.8	92.0	72.7	66.7	63.6	52.0	70.0	57.1
	hypo	28.0	7.4	9.1	48.0	33.3	10.5	14.8	11.5	30.8	26.1	4.5	21.7	25.0	8.0	22.7	33.3	36.4	44.0	25.0	42.9
Tail	raised	28.0	44.4	40.9	12.0	25.0	27.0	29.6	30.8	34.6	47.8	31.8	30.4	41.7	36.0	40.9	61.9	63.6	28.0	15.0	57.1
	Elongated	64.0	51.9	59.1	80.0	70.8	73.0	70.4	69.2	53.8	52.2	63.6	65.2	50.0	64.0	59.1	33.3	31.8	68.0	80.0	33.3
	Drag	8.0	3.7	0.0	8.0	4.2	0.0	0.0	0.0	11.5	0.0	4.5	4.3	8.3	0.0	0.0	4.8	4.5	4.0	5.0	9.5
Grip strength	strong	0.0	0.0	0.0	0.0	0.0	0.0	0.0	0.0	0.0	0.0	0.0	0.0	0.0	0.0	0.0	0.0	0.0	0.0	0.0	0.0
	normal	96.0	85.2	90.9	100	88.5	87.0	86.4	100.0	88.5	87.0	86.4	100.0	79.2	84.0	68.2	81.0	59.1	44.0	50.0	57.1
	weak	4.0	14.8	9.1	0.0	11.5	13.0	13.6	0.0	11.5	13.0	13.6	0.0	20.8	16.0	31.8	19.0	40.9	56.0	50.0	42.9
Swim ability	tumbling	0.0	0.0	0.0	0.0	0.0	0.0	0.0	3.8	0.0	0.0	0.0	0.0	0.0	0.0	0.0	0.0	0.0	0.0	0.0	0.0
	immotile	16.0	7.4	4.5	8.0	4.2	10.5	3.7	19.2	0.0	0.0	0.0	17.4	8.3	4.0	4.5	4.8	4.5	0.0	0.0	4.8
	irregular	40.0	29.6	40.9	32.0	16.7	28.9	3.7	46.2	7.7	13.0	9.1	26.1	20.8	28.0	18.2	19.0	18.2	8.0	15.0	19.0
	normal	44.0	63.0	54.5	60.0	79.2	60.5	92.6	30.8	92.3	87.0	90.9	56.5	70.8	68.0	77.3	76.2	77.3	92.0	85.0	76.2

### Astrocytic *Adrb2* does not affect motor ability

Balance and motor skills were assessed using the swim ability and rotarod tasks. At 2 and 4 weeks post induction the FLX group performed less well during the swim ability test, however this difference did not persist over time (Table 1). The rotarod test revealed that females performed better than males, a common finding in normal mice, and that all groups of mice had a shorter latency to fall as they grow older. The decline with age was not influenced by mouse genotype however, and no significant differences between the groups were noted at any of the different time points tested (Fig 2E and 2F).

### Spatial learning and memory is impaired in aged animals

**Fig 3:**
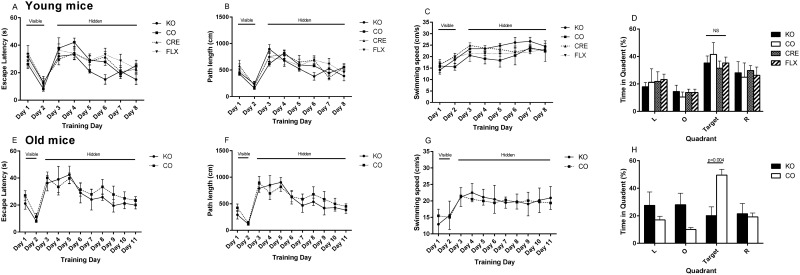
Learning phenotype of young and old *Adrb2*^*flo*x^ GLAST-CreERT2 double mutants. Escape latency (A) path length (B) and swimming speed (C) for 12 week old (n = 5–7) and aged (E, F, G respectively) animals (n = 4–6). Preference for quadrants left (L), right (R), opposite (O) of the target during the 30 second probe trial 2hours after the completion of the maze training are shown for the 12 week old (D) and aged animals (G). Graphs show mean +/- SD. The statistics for a difference between the genotypes (only) is shown. * = p<0.05, ** = p<0.01, *** = p<0.001.

Spatial learning and memory was assessed in mice 4 weeks post tamoxifen induction and in aged animals using the Morris Water Maze. In the younger animals, escape latency analysis (two-way ANOVA, genotype, day) for visible platform revealed a significant effect of trial day (F = 36.46; p < 0.0001) but no effect of genotype or of genotype-by-day interaction This indicates that the KO animals and all control groups were equally efficient at learning the non-spatial aspects of the water maze task (Fig 3A). Similar results for the path length supported that result (trial day, F = 24.52; p<0.0001). An effect of the training day was observed on swimming speed (F = 6.61; p = 0.0140) as the animals learned the task.

In the hidden platform setup, escape latency analysis (two-way ANOVA) revealed that genotype influenced learning (F = 4.31; p = 0.0064) with *Adrb2* KO mice finding the platform slightly quicker than the control groups during the training. The training day was also highly significant on the escape latency as expected (F = 7.91; p<0.0001). The path length was likewise influenced by training day (F = 5.34; p = 0.0002), but not by genotype. Swimming speed was not affected by the day in this phase of the training but some effect was attributable to genotype (F = 7.36; p = 0.0001) (Fig 3C).

Retention of spatial search strategies was evaluated in these young mice in a probe trial performed 2 h after the final hidden platform training session. Two-way ANOVA revealed no genotype-by-target quadrant interactions (F = 0.6954; p = 0.6954 and comparing the time spent in the target quadrant found no differences between the groups (one way ANOVA, F = 0.4861, p = 0.6954), demonstrating that the *Adrb2* KO mice perform as well as the controls in this spatial learning task at 4 weeks post induction. (Fig 3D).

When spatial memory was assessed in the aged animals (52–61 weeks post induction), escape latency analysis (two-way ANOVA, genotype, day) for visible platform phase revealed no significant genotype-by-day interaction or significant main effect of genotype), but a significant main effect of trial day (F = 20.06; p = 0.0004). This indicated that the aged KO and CO animals were equally efficient at learning the non-spatial aspects of the water maze task (Fig 3E). The result for the path length also indicated that both groups of animals learned the task (trial day (F = 15.53; p < 0.0010) (Fig 3F). As in the younger mice, swim velocity was not affected by genotype, trial day or the interaction between the two (Fig 3G).

In the hidden platform setup, escape latency analysis (two-way ANOVA, genotype, training day) revealed no effect of genotype but highly significant effect of training day (F = 3.37, p = 0.0024) demonstrating that both KO and CO mice were capable of learning the spatial memory task in the water maze setup (Fig 2E). No interaction between training day and genotype was observed. Path length showed similar results (training day F4.51, p<0.0001) (Fig 3F). Swimming speed of the mice was not affected by genotype day or the interaction of those factors giving us confidence that despite the different body weights of the CO and KO groups, there was not a difference in the non-spatial aspects of our Morris water maze experiment. (Fig 3G).

Retention of spatial search strategies was evaluated in aged mice in a probe trial performed 2 h after the final hidden platform training session. Two-way ANOVA showed a significant interaction between genotype and quadrant (F = 7.99, p = 0.0004). Comparing the percentage of time spent by each group in the target quadrant showed a significant difference (p = 0.0038) between the *Adrb2* KO (20.1+/-12.9%) and CO (49.6+/-10.23%) mice, indicating that while the CO mice still showed appropriate spatial learning, the aged *Adrb2* KO mice had not learnt the task. (Fig 3H).

### Long term potentiation is altered in aged animals

Because spatial memory was impaired in aged mice as observed in the Morris water maze, we tested whether long-term potentiation (LTP) could be induced and maintained over time in 6 and 18 month-old *Adrb2* KO mutant mice compared to control mice.

We first tested LTP in 6 month old KO and control mice in typical aCSF which contains glucose at a concentration of 10 mM. We found that that there were no differences in the maximal basal synaptic response (Fig 3A) between the KO and the control mice. Likewise, KO mice had equal potentiation in response to a 100 Hz stimulation.

**Fig 4:**
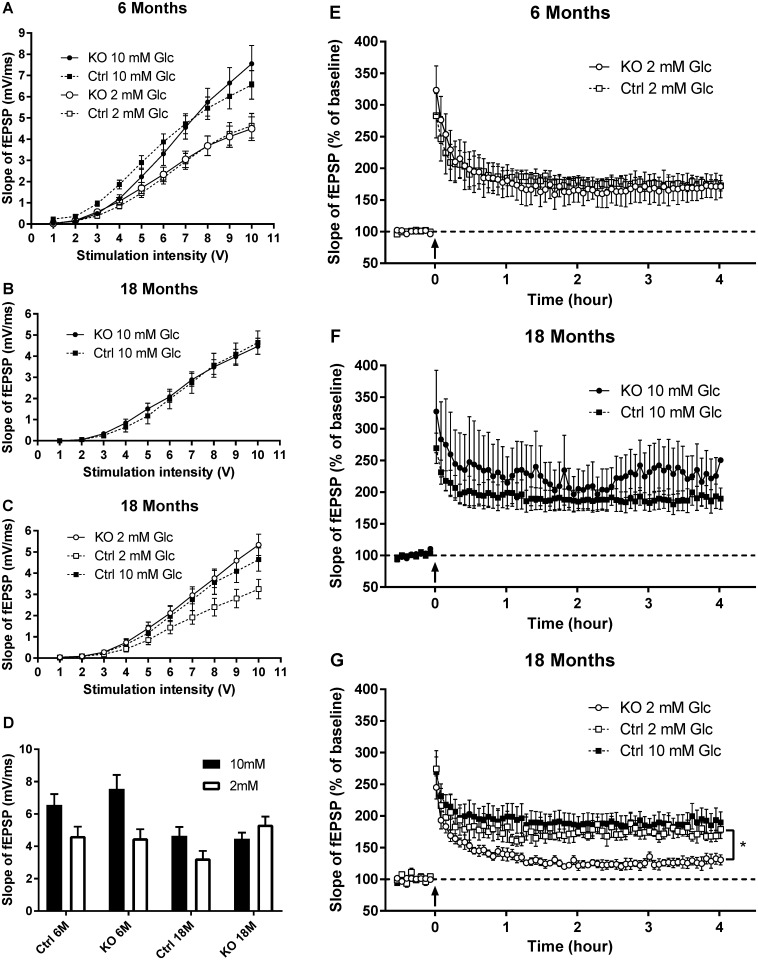
Long term potentiation results in young and aged *Adrb2*^*flo*x^ GLAST-CreERT2 double mutants in high (10 mM) and low (2 mM) glucose media. Basal synaptic conduction (A) was reduced for both KO and CO mice in the 2 mM glucose condition compared to 10 mM glucose. Both KO and CO mice maintained LTP (E) over the trial period. 18 month old mice also had similar basal synaptic conduction (B) and LTP (F) to controls in the 10 mM glucose media. In low glucose condition 18 month old KO mice did not significantly reduce their basal synaptic transmission (C) and LTP (G) was reduced. KO = *Adrb2*^*flo*x +/+^ GLAST-CreERT2^+/-^ animals treated with tamoxifen or CO = the corn oil vehicle (CO). Mean +/- SEM is displayed. The slopes of the cEPSP data are summarized in (D). The Input/output curves: Young mice 2mM glucose KO N = 3 mice and 26 slices, CO N = 3 mice and 29 slices 10mM glucose KO = 3mice and 14 slices, CO = 3 mice and 11 slices. Old mice 2mM glucose KO N = 4 mice and 12 slices, CO N = 3 mice and 12 slices 10mM glucose KO = 3mice and 10 slices, CO = 3 mice and 10 slices. LTP: Young mice 2mM glucose KO N = 3 mice and 26 slices, CO N = 3 mice and 29 slices. 10mM glucose not done. Old mice 2mM glucose KO N = 3 mice and 6 slices, CO N = 3 mice and 4 slices 10mM glucose KO = 3mice and 4 slices.

However, as learning is process that is very energy dependant, and the extracellular concentration of glucose in the hippocampus is approximately 0.2 mM [21, 22], the standard aCSF concentration of 10 mM may not provide a glucose challenge similar to that experienced during learning *in vivo*. Glucose at 2 mM in aCSF can maintain normal (murine optic nerve) axon health for several hours, whereas concentrations below 2 mM lead to a rapid loss of action potential [23] we therefore tested if LTP was maintained in the presence of 2 mM of glucose in 6 month old mutant mice. The reduction of glucose concentration from 10 mM to 2 mM induced a decrease of the basal synaptic transmission in both groups of mice. The maximal response decreased from 6.5 ± 0.7 mV to 4.6 ± 0.6 mV in control mice (n = 11) and from 7.5 ± 0.9 mV to 4.6 ± 0.6 mV (n = 14) in mutant mice (Fig 4A). After one train of stimulation at 100 Hz, the level of potentiation reached in mutant mice was not different from that observed in WT mice in the same conditions (Fig 4E). Moreover, the potentiation was maintained in both groups for 4 hours (172 ± 8% and 171 ± 18% four hours after the train in control (n = 4) and KO mice respectively (n = 3).

In aged (18 month old) mice, similarly to the 6 month old mice, no difference was observed between KO and control mice when 10 mM glucose was used for slice incubation. Basal synaptic transmission, presented in Fig 4B, was not different with a maximal response of 4.6 ± 0.5 mV in control and 4.5 ± 0.4 mV in KO mice (n = 10, t test, p = 0.8). Long-term potentiation induced in mutant mice by 1 train at 100 Hz was maintained at 225 ± 29% (n = 5) for 4 hours, which was not different from the value measured in control mice (193 ± 19%, n = 4, t test p = 0.37, Fig 4F).

When the aCSF glucose concentration was reduced from 10 mM to 2 mM, this relative glucose deprivation induced a decrease in the basal synaptic response in control mice from 4.6 ± 0.5 mV to 3.2 ± 0.4 mV (Fig 4C, t test, p<0.05). By contrast, the basal synaptic response in the aged *Adrb2* KO mice was not modified with a maximal value of 5.3 ± 0.5 mV (Fig 4C, t test, p = 0.2). In the presence of 2 mM glucose, the basal synaptic transmission was statistically different between mutant and control mice (t test, p<0.01).

LTP was induced by one train at 100 Hz in the presence of 2 mM glucose. The initial level of potentiation was the same in both groups of aged mice (274 ± 29% and 245 ± 18% for CO (n = 4) and *Adrb2* KO mice (n = 6) respectively). However, if the potentiation was maintained in control mice at the same level as that observed with 10 mM glucose (193 ± 19%), it rapidly decreased in *Adrb2* KO mice. Fig 4G shows that the final level of potentiation was 179 ± 14% in WT and 131 ± 8% in mutant mice (t test, p<0.05). The slopes of the cEPSP data are summarized in (4D).

### White matter extracellular glucose and lactate

**Fig 5:**
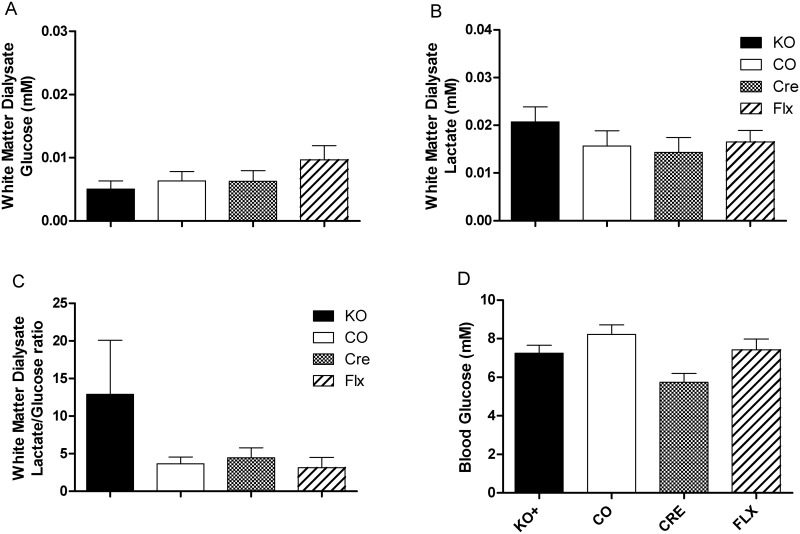
Central glucose and lactate and peripheral glucose in *Adrb2*^*flo*x^ GLAST-CreERT2 double mutants and controls. Concentration of (A) glucose and (B) lactate and the (C) lactate/glucose ratio from microdialysate collected from the cerebellar white matter. (D) Blood glucose in mice fasted for 6 hours. For microdialysis results N = 10–14 for each genotype, 3 biological replicates. Blood glucose are N = 10–12 for each genotype, excluding CRE (N = 3).

As the β2AR can influence glycogenolysis and energy availability in the CNS, we tested the extracellular glucose and lactate concentrations in the CNS. We did not observe any significant effect of genotype on the glucose concentration in the dialysate collected from the cerebellar white matter of free living mice at 4 weeks after the tamoxifen induction (Fig 5A). Likewise, there was no difference observed in the lactate concentration of those same dialysate samples (Fig 5B).

When the ratio of lactate and glucose was assessed, (mM lactate/mM glucose) while there were no significant differences between the four groups, the results were suggestive of an increased relative amount of lactate in the dialysate of the KO mice relative to the other groups (Fig 5C). While this difference wasn’t significant (F = 1.724, p = 0.1675), the variance was significantly different between groups (Bartlett’s statistic = 117.6, p<0.0001) seemingly due to the KO group.

### Blood glucose

As the brain also takes up glucose from the circulating blood, we assessed glucose levels in the peripheral blood of mice that were fasted for 5–6 hours prior to measurement (Fig 5D). No differences were found in the blood glucose levels of male and female mice of each genotype, and so the results of the sexes were combined (N = 7–13). No differences were observed between the different groups, from which we inferred that altered levels of circulating blood glucose were not key to the effects on memory associated with the loss of β2AR on astrocytes.

## Discussion

In this paper we describe the effects of eliminating the expression of β2AR in GLAST-expressing astrocytes in mice. The main results seen in this model were related to spatial memory tasks and neuroplasticity in brain slices taken from aged mice. Aged KO mice performed less well on the water maze task compared to control mice indicating that there was impairment in their spatial memory formation. Likewise, LTP was impaired in those mice. Interestingly, uniquely the aged KO mice did not reduce basal synaptic activity in response to a reduced glucose in the artificial CSF media. In contrast to the aged mice, there were no differences to any of these parameters seen in 6 month old mice where the KO mice performed as well as the mice.

There is a well-known inverted U-shaped relationship between NE signaling and the enhancement of memory formation [24]. NE is synthesized in the *locus coeruleus* and noradrenergic nerve fibers span the forebrain, potentially affecting the function of all cells types [2, 4]. Experiments using hippocampal slice cultures have also shown that β-AR signaling is essential for generating LTP. In the presence of β-AR antagonist, both electrically induced and learning-facilitated LTP is blocked [25]. Further to this, the injection of β-AR antagonists into the CA1 region of the hippocampus impairs long-term memory in a water maze task [26].

Previous studies have hypothesized on how βAR signaling could be affecting memory formation and LTP. Noradrenergic nerve fibers express adrenoreceptor subtypes pre- and post-synaptically and thus it makes sense that direct NE signaling in those cells would affect LTP. For instance, it is known that NE signaling in neurons drives phosphorylation of GluR1 to facilitate AMPA receptor trafficking to the synapse [27] However, in this body of work, β2AR expression was removed from astrocytes (not neurons) and deficiencies in memory formation were still observed. Thus, while an important mechanism of β-AR signaling has an immediate effect on memory formation and is likely to be present in neurons, another mechanism that is found in astrocytes must also be involved, at least in aged animals.

One mechanism by which NE and β2AR signaling might be affecting memory formation via the astrocytes is the effect of noradrenergic signaling on glycogenosis and the availability of energy substrates in the extracellular fluid. In the brain, astrocytes are the major, if not the only storage site of glycogen, and the evidence is that glycogenolysis in astrocytes supports memory, as pharmaceutically blocking glycogenolysis via intracerebral injection inhibits memory consolidation [28]. The increased demand for extracellular glucose in the brain itself during memory formation is also well demonstrated. More specifically, injections of glucose into specific regions of the brain lead to enhanced cognitive performance [29]. Likewise lactate, a product of astrocytic glycogenolysis, supports memory formation [30]. Under baseline conditions, the uptake of glucose by astrocytes and neurons is approximately equal, but when neocortical activity is stimulated by manipulating the whiskers, glucose is preferably taken up by astrocytes [31]. In training tasks where there is a lot of demand, the extracellular glucose levels in rats can reduce by 30–40%, and lactate levels increase [30, 32]. Experiments including inhibition of the lactate export from the astrocytes by monocarboxylate transporter (MCT) 4 or 1, or neuronal import of lactate by MCT2, impede memory and LTP formation [33]. Providing exogenous L-lactate rescued the amnesiac phenotype in MCT4 and 1 disruption, but not when MCT2 was disrupted, suggesting that L-lactate import by neurons is essential for developing LTP and spatial working memory [30, 33]. The situation is complicated however, as aspects of brain function other than learning are also affected by β2AR signaling. For instance, signaling through the receptor improves axonal protection after prolonged aglycaemia and reperfusion via a mechanism that is may not be related to white matter lactate levels [34].

We assessed the extracellular glucose and lactate energy substrates in the white matter in mice 4 weeks post tamoxifen induction. There were no significant differences in extracellular glucose or lactate concentration between the genotypes. Likewise, there was no significant difference in the lactate/glucose ratio. However, there was a substantial increase in the variation of the ratio in the KO. One might hypothesize that the responsiveness to energy demand is altered in the aged KO mice. In this battery of experiments, we did not assess the aged mice with microanalysis due their relative frailty, however it would be interesting to assess extracellular CNS glucose and lactate levels in the hippocampi of these mice during cognitive tasks.

A question remains as to why the effects on spatial memory development, LTP and basal synaptic transmission were present in aged mice but not in younger mice that had the same astrocytic β2AR expression deficit. Aged mice show substantially larger decreases in hippocampus extracellular glucose levels during training, which is associated with cognitive impairments compared to young mice. That age related impairment, along with the marked drop in extracellular glucose can be ameliorated if the animal is given a systemic bolus of glucose before the training [35]. In contrast, lactate is markedly higher in the extracellular fluid of the brain in aged animals compared to younger animals, and in humans the increase of lactate in response to stimulus is much reduced or absent in old age, suggesting that the metabolic processes of the brain are disturbed as time goes on [36, 37] and are similar to a state of “high demand” in younger mice. It may be that the energy metabolism in the aged mice is at the limit of functionality, potentially rendering the contribution of β2AR signaling more important than in younger mice. Further studies are required to better determine the underlying mechanisms.

## Supporting Information

S1 Fig β2AR mRNA expression in CNS cell—enriched populations collected by laser capture dissection four weeks post tamoxifen administration. (DOCX)

S2 Fig Learning phenotype of aged ADRB2 control mice at two ages. (DOCX)

S1 Methods Supporting methods. (DOCX)
