# Supplementary material for: Astrocytic β2 Adrenergic Receptor Gene Deletion Affects Memory in Aged Mice
Source: PLoS One. 2016 Oct 24;11(10):e0164721. doi: 10.1371/journal.pone.0164721 (PMC5077086; doi:10.1371/journal.pone.0164721)
Supplement: S1 Fig — (DOCX) [file pone.0164721.s001.docx]

Supporting information for “Astrocytic β2 adrenergic receptor gene deletion in mice affects memory in aged mice”. Jensen et al.

S1 Figure: β2AR mRNA expression in CNS cell – enriched populations collected by laser capture dissection four weeks post tamoxifen administration. Approx. 1500 cells per animal, N=3 per group. All samples are flox +/+.

The expression of β2AR is known to be different in different subtypes of neuron and to alter with activation state of microglia ([Gyoneva and Traynelis 2013](#_ENREF_1)). We believe that this may explain some of the heterogeneity of expression in the NeuN+ and Iba+ groups.

Gyoneva, S. and S. F. Traynelis (2013). "Norepinephrine modulates the motility of resting and activated microglia via different adrenergic receptors." J Biol Chem **288**(21): 15291-15302.
