# Supplementary material for: Astrocytic β2 Adrenergic Receptor Gene Deletion Affects Memory in Aged Mice
Source: PLoS One. 2016 Oct 24;11(10):e0164721. doi: 10.1371/journal.pone.0164721 (PMC5077086; doi:10.1371/journal.pone.0164721)
Supplement: S2 Fig — (DOCX) [file pone.0164721.s002.docx]

Supporting information for “Astrocytic β2 adrenergic receptor gene deletion in mice affects memory in aged mice”. Jensen et al.


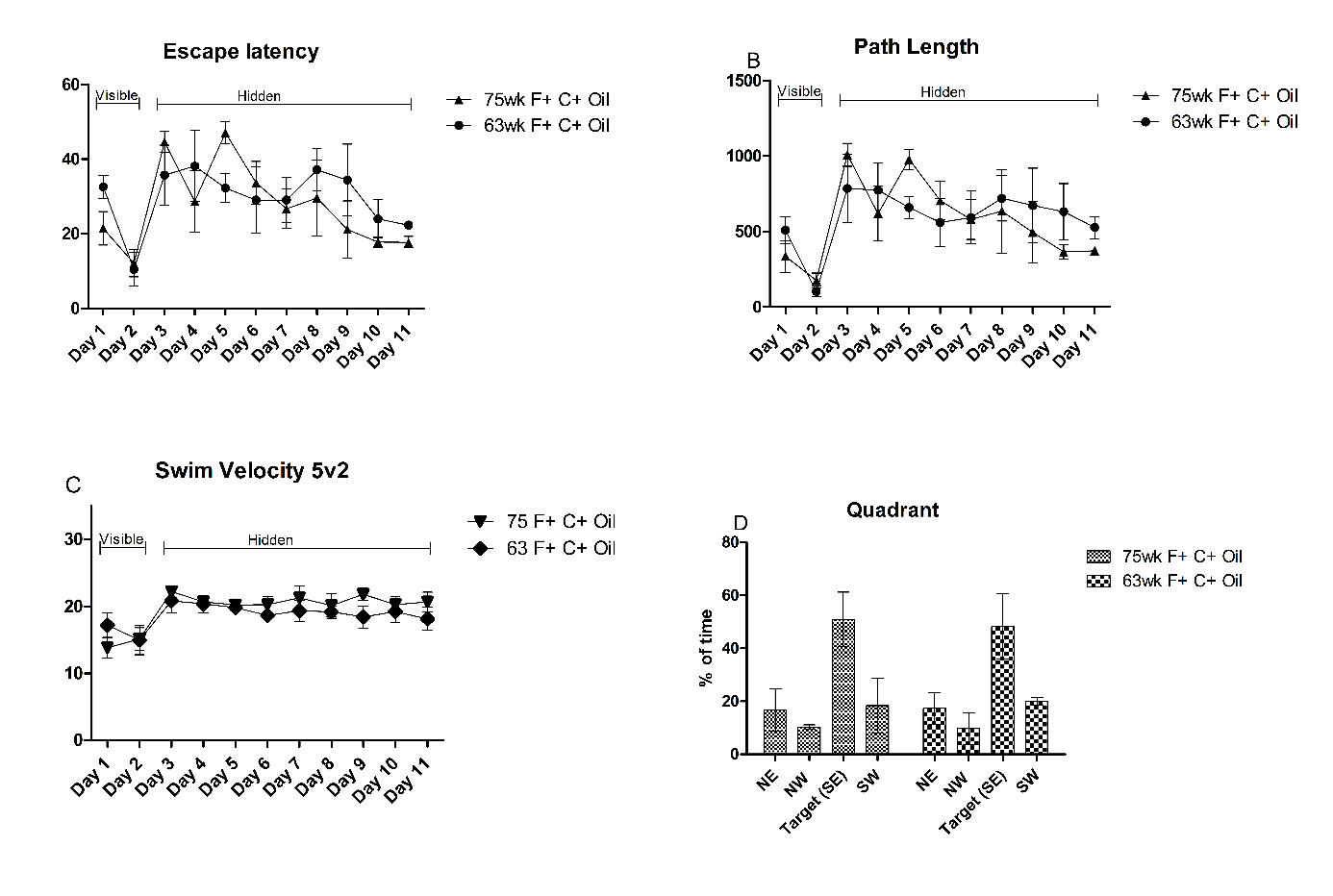


Swim speeed (cm/sec)

Path Length (cm)

Escape latency (s)

S2 Fig: Learning phenotype of aged ADRB2 control mice at two ages. Escape latency (A) path length (B) and swimming speed (C) for aged Flox+/+ (F+) Cre +/- (Cre+) mice at 75 and 63 weeks of age. Preference for quadrants to the left (NE), right (SW) or opposite (NE) of the target (SE) during the 30 second probe trial 2hours after the completion of the maze training are also shown (D).
