# Supplementary material for: Astrocytic β2 Adrenergic Receptor Gene Deletion Affects Memory in Aged Mice
Source: PLoS One. 2016 Oct 24;11(10):e0164721. doi: 10.1371/journal.pone.0164721 (PMC5077086; doi:10.1371/journal.pone.0164721)
Supplement: S1 Methods — (DOCX) [file pone.0164721.s003.docx]

Supporting information for “Astrocytic β2 adrenergic receptor gene deletion in mice affects memory in aged mice”. Jensen et al.

**Supplementary methods**

Laser capture microscopy. Tissue was cut in 10µm sections and mounted on uncharged microscope slides and stored at -80◦C until use. Slides were fixed in -20◦C acetone and the allowed to dry then fixed and dried again. Tissue was treated with 1.5% H_2_O_2_ in TBS and then washed in TBS, then blocked with 10% inactivated FBS. Slides were stained with anti-GFAP (Dako Z0334), Anti-Iba1 (Wako 019–19741) or anti-NeuN (Millipore MAB377) and appropriate secondary antibodies. The desired tissue was collected with disposables from the Arcturus LCM system (Thermofisher Scientific) used with CapSure Macro LCM caps. The RNA was isolated using the Picopure RNA isolation kit.

Staining. All steps were performed as RNase-free as possible. Sectioned tissue mounted on uncharged microscope slides was fixed in acetone at -20^◦^C for 5 minutes. The endogenous peroxidases were quenched with 1.5% H_2_O_2_ and the sample washed in TBS for 5 seconds. Tissue was blocked and then stained with the appropriate antibody for 10 minutes (GFAP (Dako Z0334), IbaI NeuN (Millipore MAB377), washed with TBS, then incubated with the biotinylated secondary antibody for 5 minutes, then washed again. The staining was treated with avidin-biotinylated horseradish peroxidise (Dako), washed and visualized with the Liquid DAB+ Substrate Chromogen System (Dako) for 5-10 minute. The tissue was then washed 3 times in TBS, then dehydrated in graduated EtOH and 100% xylene. The slides were allowed to dry, and then stored in a desiccator until laser capture microdissection.
